# Assessment of AOPP, TBARS, and Inflammatory Status in Diabetic Nephropathy and Hemodialyzed Patients

**DOI:** 10.3390/ijms262110670

**Published:** 2025-11-01

**Authors:** Daniel Cosmin Caragea, Lidia Boldeanu, Mohamed-Zakaria Assani, Mariana-Emilia Caragea, Alexandra-Ștefania Stroe-Ionescu, Romeo Popa, Daniela-Teodora Maria, Vlad Pădureanu, Cristin Constantin Vere, Mihail Virgil Boldeanu

**Affiliations:** 1 Department of Nephrology, Faculty of Medicine, University of Medicine and Pharmacy of Craiova, 200349 Craiova, Romania; daniel.caragea@umfcv.ro (D.C.C.); daniela.maria@umfcv.ro (D.-T.M.); 2 Department of Microbiology, Faculty of Medicine, University of Medicine and Pharmacy of Craiova, 200349 Craiova, Romania; lidia.boldeanu@umfcv.ro; 3 Doctoral School, University of Medicine and Pharmacy of Craiova, 200349 Craiova, Romania; alexandra.stroe@yahoo.com; 4 Department of Immunology, Faculty of Medicine, University of Medicine and Pharmacy of Craiova, 200349 Craiova, Romania; mihail.boldeanu@umfcv.ro; 5 Department of Pharmacology, University of Medicine and Pharmacy of Craiova, 200349 Craiova, Romania; romeo_rop@yahoo.com; 6 Department of Internal Medicine, University of Medicine and Pharmacy of Craiova, 200349 Craiova, Romania; vlad.padureanu@umfcv.ro; 7 Department of Gastroenterology, University of Medicine and Pharmacy of Craiova, 200349 Craiova, Romania; vere_cristin@yahoo.com

**Keywords:** oxidative stress, inflammation, AOPP, TBARS, diabetic nephropathy, hemodialysis, chronic kidney disease

## Abstract

We compared oxidative markers and their links to inflammation in diabetic nephropathy and hemodialysis to identify independent determinants. We studied 180 adults, 90 patients with type 2 diabetes and diabetic nephropathy and 90 patients on hemodialysis. We measured serum advanced oxidation protein products (AOPP) and thiobarbituric acid reactive substances (TBARS) by enzyme-linked immunosorbent assay (ELISA). Partial correlations were adjusted for age, sex, and albumin with false discovery rate (FDR) control. Principal component analysis (PCA) summarized inflammatory indices and linear models tested predictors of AOPP and TBARS. Oxidative damage was higher in hemodialysis, with AOPP median 25.80 versus 5.06 and TBARS 8.49 versus 1.89, *p* less than 0.0001. C reactive protein (CRP) and mean corpuscular volume-to-lymphocyte ratio (MCVL) were higher in patients ongoing hemodialysis; systemic immune-inflammation index (SII) was higher in diabetic nephropathy. PCA yielded a dominant inflammation axis in both cohorts, 74.73 percent in hemodialysis and 85.20 percent in diabetic nephropathy. In regression, creatinine (β = 2.47, *p* = 0.026) predicted AOPP in hemodialysis. Dialysis vintage inversely predicted TBARS, β = −0.2305, *p* = 0.0209. In diabetic nephropathy, the PCA inflammation score predicted AOPP, β = 1.134, *p* = 0.0003. Protein oxidation tracked systemic inflammation in diabetic nephropathy, but not in hemodialysis. AOPP outperformed TBARS as an inflammatory partner and a practical monitoring candidate in diabetic kidney disease. Prospective studies should test for prognostic value and therapy sensitivity.

## 1. Introduction

Kidney pathology represents a critical public health concern worldwide, with diagnosis frequently occurring at advanced stages characterized by elevated serum creatinine and urea levels. Prior to the manifestation of overt clinical symptoms, molecular perturbations within renal tissue, particularly within proximal tubular cells densely populated with mitochondria, may be detectable. Mitochondria, as primary sources of reactive oxygen species (ROS), render renal parenchyma especially vulnerable to oxidative insult. During episodes of both acute and chronic renal injury, an overproduction of ROS and other pro-oxidants can exacerbate tissue damage and precipitate secondary pathologies [1].

Diabetic nephropathy (DN) remains one of the most serious microvascular complications of diabetes mellitus and a leading cause of end-stage renal disease worldwide. Chronic hyperglycemia causes multiple metabolic and hemodynamic disturbances, which lead to oxidative stress, endothelial dysfunction, inflammation, and glomerular damage in DN patients. Recent reviews highlight that oxidative stress and the related inflammation create a vicious cycle that causes renal cell injury, glomerulosclerosis, tubular atrophy, and fibrosis in diabetic kidneys [2].

Oxidative stress refers to an imbalance between the production of ROS and the body’s antioxidant defenses. In DN, ongoing high blood sugar levels increase ROS generation via mechanisms such as mitochondrial overload, activation of nicotinamide adenine dinucleotide phosphate (NADPH) oxidase, formation of advanced glycation end products (AGEs), and stimulation of protein kinase C (PKC) [3]. Elevated ROS leads to lipid peroxidation, protein oxidation, deoxyribonucleic acid (DNA) damage, and activates proinflammatory pathways like nuclear factor kappa-light-chain-enhancer of activated B-cells (NF-κB). Some researchers suggest that endoplasmic reticulum (ER) stress and mitochondrial dysfunction also play roles in linking oxidative stress to diabetic kidney damage [3,4]. Patients with chronic kidney disease (CKD) face ongoing oxidative stress and systemic inflammation, which not only hasten the decline of renal function but also elevate the risk of cardiovascular morbidity and mortality [5].

Patients with advanced diabetic nephropathy may need dialysis, such as hemodialysis or peritoneal dialysis. During these treatments, oxidative stress typically increases due to factors like bioincompatibility of the dialysis membrane, endotoxins in dialysate, uremic toxin buildup, and ongoing inflammation. Those on hemodialysis often face more oxidative damage to proteins and higher levels of lipid peroxidation products. These oxidative stresses contribute to cardiovascular problems, endothelial dysfunction, atherosclerosis, and further damage to the kidneys [6,7].

Advanced oxidation protein products (AOPP) indicate oxidative changes in plasma proteins, mainly albumin, caused by chlorinated oxidants. Elevated AOPP levels have been linked to inflammation and vascular damage in cases of renal injury. AOPP have been demonstrated to promote inflammation by stimulating ROS production and activating macrophages, potentially contributing to tissue damage in CKD. Elevated levels of AOPP are consistently found in patients with CKD and those on dialysis, with levels tending to rise as kidney function declines. In fact, dialysis patients typically exhibit higher AOPP levels than CKD patients who are not on dialysis [8,9,10,11,12].

Thiobarbituric acid reactive substances (TBARS) are commonly used as an indicator of lipid peroxidation in biological systems, signifying damage to membrane lipids during oxidative stress. In population research, TBARS correlate positively with blood glucose levels and negatively with antioxidant defenses such as glutathione, confirming its role as a broad marker of redox imbalance [13,14].

Our study aimed to assess the levels of AOPP and TBARS in patients with diabetic nephropathy and those on dialysis. The specific objectives included:

I. To compare AOPP and TBARS values between the two patient groups.
II. To analyze correlations between AOPP, TBARS, and selected clinical and biochemical indices.
III. To assess the potential role of these oxidative stress markers as indicators of disease severity and progression.

## 2. Results

### 2.1. Demographic and Clinical Characteristics

Table 1 presents the demographic, anthropometric, hematological, renal, and biochemical parameters of the study population. These data were analyzed to compare baseline characteristics between the hemodialysis (HD) and type 2 diabetes mellitus with diabetic nephropathy (T2DM-DN) groups and to identify distinct metabolic and clinical patterns associated with each condition.

The median age was significantly higher in the T2DM-DN group compared with the HD group (67 vs. 61 years, *p* = 0.03). The sex distribution did not differ significantly between groups (*p* = 0.454). A higher proportion of T2DM-DN patients lived in urban areas compared with HD (*p* < 0.0001). The T2DM-DN group had significantly higher body weight and BMI than the HD group (*p* < 0.0001 for both). Height was comparable between groups (*p* = 0.281).

White blood cell (WBC), neutrophil (NEU), lymphocyte (LYM), and platelet (PLT) counts were significantly higher in the T2DM-DN group (*p* < 0.0001 for all but LYM, with a *p* of 0.0005). Basophil (BAS) and eosinophil (EOS) counts were lower in HD patients (*p* = 0.0006 and *p* = 0.012, respectively). Red blood cell (RBC) count, hematocrit (HCT), and hemoglobin (HGB) levels were significantly higher in T2DM-DN (*p* < 0.0001 for RBC and HGB, and a *p* of 0.0003 for HCT). Mean corpuscular volume (MCV) was higher in HD patients (*p* < 0.0001).

Serum creatinine and urea levels were markedly higher in HD patients (*p* < 0.0001 for both). The estimated glomerular filtration rate (eGFR) was significantly lower in HD (median 5.51 vs. 57.39 mL/min/1.73 m^2^, *p* < 0.0001), consistent with severe renal impairment in this group. Serum sodium levels did not differ significantly between groups (*p* = 0.198). Potassium levels were slightly higher in HD (*p* = 0.006), while chloride was also elevated in HD (*p* < 0.0001). Alanine transaminase (ALT) and aspartate aminotransferase (AST) activities were significantly higher in the T2DM-DN group (*p* = 0.001 and *p* < 0.0001, respectively). There were no significant differences in triglyceride (TG) or total cholesterol (TC) levels between HD and T2DM-DN groups (*p* = 0.668 and *p* = 0.777, respectively).

The T2DM-DN group exhibited higher BMI, inflammatory cell counts, and liver enzyme activity, reflecting a metabolic and inflammatory burden. The HD group showed severe renal dysfunction characterized by elevated creatinine and urea levels, low eGFR, and electrolyte imbalances.

### 2.2. Levels of AOPP and TBARS and Inflammatory Status

Table 2 summarizes systemic inflammation and oxidative stress markers in HD and T2DM-DN patients. These indicators were assessed to determine the balance between inflammatory activity, oxidative damage, and immune cell response, with emphasis on AOPP, TBARS, and leukocyte parameters, such as the ratio between mean corpuscular volume and lymphocytes (MCVL).

C-reactive protein (CRP) was significantly higher in the HD group compared with T2DM-DN (median 4.50 vs. 1.08 mg/dL, *p* < 0.0001), indicating a stronger systemic inflammatory response in hemodialysis patients.

Albumin (ALB) levels were lower in HD patients (*p* < 0.0001), consistent with chronic inflammation and protein-energy wasting commonly observed in end-stage renal disease. The inflammatory indices AISI, SIRI, NPR, NLR, and PLR showed no significant differences between groups (*p* > 0.05). However, SII was significantly elevated in T2DM-DN (*p* = 0.002), suggesting a higher inflammatory burden related to metabolic dysregulation.

Among leukocyte-derived ratios, the monocyte-to-lymphocyte ratio (MLR) and MCVL differed markedly between groups. MCVL was significantly higher in HD patients (median 61.42 vs. 46.28, *p* < 0.0001). This finding indicates altered leukocyte activation and volume regulation in chronic uremic conditions, likely linked to persistent oxidative and osmotic stress. MLR was lower in T2DM-DN (*p* < 0.0001), suggesting different immune cell dynamics compared with HD.

AOPP and TBARS, both markers of oxidative damage, were markedly higher in HD patients (AOPP: 25.80 vs. 5.06, TBARS: 8.49 vs. 1.89, both *p* < 0.0001). These results demonstrate a strong oxidative stress state in hemodialysis, reflecting protein and lipid oxidation caused by repeated exposure to bioincompatible dialysis membranes and uremic toxins.

Overall, HD patients exhibited pronounced oxidative stress, reflected by elevated AOPP and TBARS, along with higher CRP and MCVL levels, which point to sustained systemic inflammation. In contrast, T2DM-DN patients showed higher SII values, suggesting inflammation driven by metabolic and vascular factors rather than uremic toxicity.

### 2.3. Partial Correlations of AOPP and TBARS and Inflammatory Status

To explore the relationship between oxidative stress and systemic inflammation while minimizing the influence of demographic and nutritional factors, partial correlation analyses were performed. The analyses assessed associations between oxidative stress markers (AOPP and TBARS) and inflammatory indices (AISI, IIC, SII, SIRI, MCVL, NLR, MLR, PLR, dNLR, NPR) in two patient groups: individuals undergoing hemodialysis and those with diabetic nephropathy.

All correlations were adjusted for age, sex, and serum ALB to account for the confounding effects of these variables on both oxidative and inflammatory parameters. The results were further corrected for multiple comparisons using the Benjamini–Hochberg false discovery rate (FDR) procedure to reduce the likelihood of type I error. The corrected partial correlation coefficients (r), unadjusted *p*-values, and FDR-adjusted q-values are presented in Table 3, below.

After controlling for age, sex, and serum albumin, distinct differences were observed between the HD and T2DM-DN groups regarding the association between oxidative stress and inflammatory indices.

In the hemodialysis group, no significant correlations remained after FDR adjustment. Although AOPP showed a weak positive association with MCVL (r = 0.238, *p* = 0.024), the relationship lost significance after correction (q = 0.489). All other correlations between oxidative markers (AOPP, TBARS) and inflammation-related indices (AISI, IIC, SII, SIRI, NLR, MLR, PLR, dNLR, NPR) were weak and nonsignificant. These results might indicate that in end-stage renal disease, once demographic and nutritional effects are accounted for, oxidative stress and inflammatory activity appear largely independent.

In contrast, the T2DM-DN group demonstrated robust and consistent positive correlations between AOPP and multiple inflammatory markers even after FDR adjustment. Moderate correlations were observed with IIC (r = 0.428, q = 0.0002), NLR (r = 0.429, q = 0.0002), MCVL (r = 0.401, q = 0.0004), and dNLR (r = 0.404, q = 0.0004), among others. The association between AOPP and composite indices such as SII and SIRI also remained statistically significant after correction (q < 0.001). However, TBARS showed no significant associations with inflammatory markers after adjustment, suggesting that protein oxidation rather than lipid peroxidation is more closely linked to systemic inflammation in diabetic nephropathy.

These findings highlight a clear divergence between disease stages: oxidative and inflammatory coupling are strong and statistically robust in diabetic nephropathy but largely absent in hemodialysis. The persistence of AOPP-related associations after FDR correction reinforces the role of protein oxidation as a central driver of inflammatory activation in early renal injury. In contrast, in advanced disease, external factors related to dialysis may disrupt this relationship.

**Figure 1:**
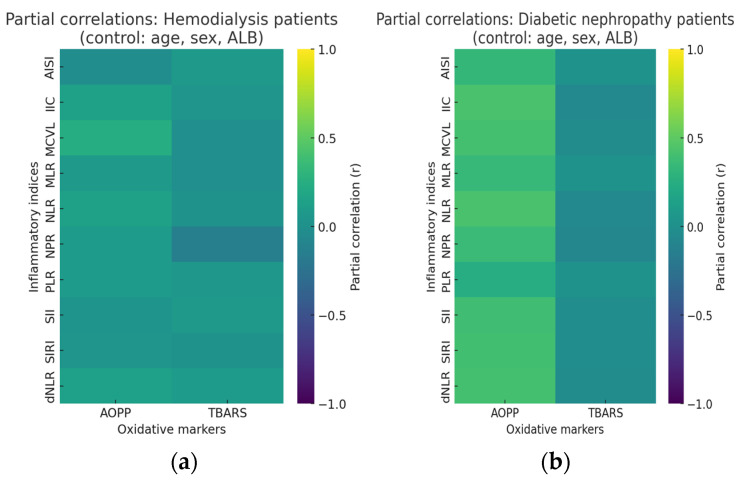
Partial correlation heatmaps between oxidative stress and inflammatory markers in diabetic nephropathy and hemodialysis patients after adjustment for age, sex, and serum ALB. The color scale represents the strength and direction of partial correlations (r), ranging from −1 to +1. In the diabetic nephropathy group, AOPP shows consistent positive correlations with several inflammatory indices, suggesting a close link between protein oxidation and systemic inflammation. In the hemodialysis group, correlations appear weaker and more variable, indicating a less stable oxidative and inflammatory relationship likely influenced by treatment-related and metabolic factors. (**a**) Partial correlations of HD patients, (**b**) partial correlations of T2DM-DN patients.

Partial correlation matrices were computed for both study groups to visualize the strength and direction of associations between oxidative stress and inflammatory markers. The analyses controlled for potential confounders, including age, sex, and serum ALB. Heatmaps were generated to illustrate the adjusted correlation coefficients between oxidative stress markers (AOPP, TBARS) and a panel of inflammatory indices (AISI, IIC, SII, SIRI, MCVL, NLR, MLR, PLR, dNLR, NPR). The color gradient represents the strength of correlation (r), ranging from −1 to +1, with warmer colors indicating stronger positive associations (Figure 1).

The heatmap for the diabetic nephropathy group shows a consistent pattern of positive correlations between AOPP and multiple inflammatory indices after controlling for age, sex, and albumin. The higher intensity of the green–yellow hues across several indices (such as IIC, NLR, and SIRI) indicates that oxidative protein damage remains closely associated with inflammatory activation in diabetic nephropathy. TBARS, however, exhibit weaker or negligible correlations, suggesting that lipid peroxidation contributes less significantly to the systemic inflammatory response in this group.

In contrast, the hemodialysis group displays a less coherent pattern, with generally weaker and more variable associations across markers. The colors remain predominantly within the low-to-mid range, reflecting modest correlations often approaching zero after adjustment. This attenuation of relationships suggests that the oxidative and inflammatory link is less stable in end-stage renal disease, likely due to the multifactorial effects of dialysis treatment, fluctuations in metabolic parameters, and broader interindividual variability.

Taken together, these visual findings align with the quantitative FDR-adjusted results, reinforcing that the coupling between oxidative stress and inflammation is more pronounced and statistically reliable in diabetic nephropathy than in hemodialysis.

### 2.4. Principal Component Analysis of Inflammatory Indices in Hemodialysis and Diabetic Nephropathy Cohorts

We performed principal component analysis (PCA) on ten inflammatory indices (AISI, IIC, SII, SIRI, MCVL, NLR, MLR, PLR, dNLR, NPR) in the two independent cohorts: patients on HD and patients with DN. The component retention was decided by parallel analysis (1000 simulations). Each cohort contributed 90 analyzable cases; no data were missing (Table 4 and Table 5).

The principal component analysis revealed a dominant first component in both groups. In HD, PC1 had an eigenvalue of 7.47, accounting for 74.73% of the variance. PC2 had an eigenvalue of 1.39, explaining 13.86%, bringing the total explained variance to 88.60% when combined. Each additional component contributed less than 7% to the total variance.

**Figure 2:**
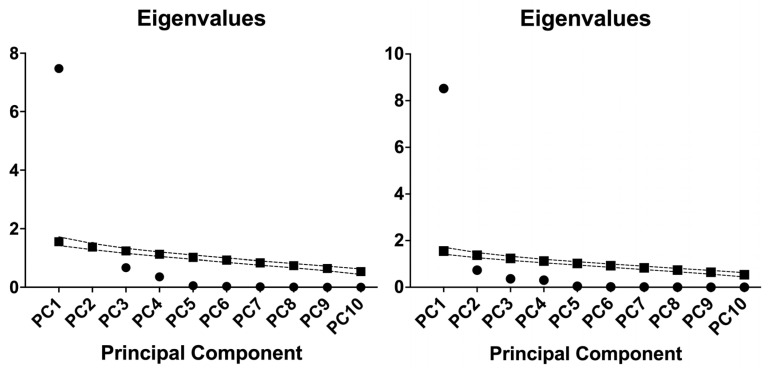
Eigenvalue scree plots for HD (**left**), T2DM-DN (**right**). Scree plots with parallel analysis for PCA of ten inflammatory indices in two cohorts. Circles: Eigenvalue (from data). Squares: Eigenvalue (from Parallel Analysis). The figure shows a clear elbow at the first component in both cohorts. Eigenvalues drop sharply after PC1. In DN, only PC1 exceeds 1.0, with 85.20% variance explained. The curve flattens from PC2 onward. In HD, PC1 and PC2 exceed 1.0. PC1 explains 74.73% and PC2 adds 13.86%. From PC3 onward, each component contributes under 7%. These scree profiles indicate a dominant general component, stronger in DN. The loading patterns support this. In DN, all indices load highly on PC1, for example, IIC −0.979, NLR −0.978, SIRI −0.965, dNLR −0.941. In HD, PC1 is also coherent, but dNLR shows a weaker loading, −0.376, and NPR is near zero, approximately 0.080. This heterogeneity in HD aligns with the presence of a small secondary component. Overall, the figure supports a primarily unidimensional structure for these indices, most clearly in DN. The HD scree curve and loadings indicate a strong first component with limited residual structure. These observations are consistent with the variance profiles reported in Table 4.

Figure 2 shows the eigenvalue scree plots with the parallel-analysis reference for each cohort, documenting the one-component solution.

In DN, PC1 had an eigenvalue of 8.52, accounting for 85.20% of the variance. No later component surpassed 7.5%. The cumulative variance was 92.52% by PC2 and 96.16% by PC3. PC1 exhibited consistent loadings across various inflammatory indices. In HD, the most significant absolute loadings were for NLR (−0.982), SII (−0.977), PLR (−0.973), IIC (−0.973), SIRI (−0.967), and MLR (−0.956). AISI (−0.942) and MCVL (−0.882) also showed strong loadings. dNLR had a modest loading of −0.376, while NPR’s loading was close to zero at almost 0.080.

In DN, all indices loaded highly on PC1. IIC and NLR had the largest absolute loadings, −0.979 and −0.978. SIRI and SII followed, −0.965 and −0.965, with dNLR at −0.941 and MLR at −0.924. AISI and MCVL loaded at −0.915 and −0.910. NPR and PLR also contributed, −0.875 and −0.757.

These patterns may indicate a common latent dimension shared by the indices in both cohorts, stronger and more uniform in DN. The negligible PC1 loading of NPR in HD and the modest loading of dNLR in HD suggest cohort specific differences in how these markers co-vary with the broader inflammatory signal.

### 2.5. Multiple Linear Regressions

#### 2.5.1. Multiple Linear Regressions of HD Cohort

##### Multiple Linear Regressions of HD Cohort for AOPP

Two predefined models were examined. Model 1 incorporated demographic and dialysis-related covariates: sex, dialysis vintage, membrane type, age, and BMI. Model 2 included laboratory and inflammation-related covariates: creatinine, albumin, the PCA inflammation score (PC-inflammation), CRP, and eGFR. All analyses employed ordinary least squares. The alternative, Model 2, was slightly favored based on the small-sample corrected Akaike Information Criterion (AICc) (probability 60.6% versus 39.4% for Model 1; ΔAICc = 0.86; likelihood ratio = 1.54), suggesting only modest evidence supporting Model 2 over the simpler model (Table 6 and Table 7).

Model 1 (clinical/dialysis covariates): Overall fit was not significant, with low explained variance (R^2^ = 0.072; F (5, 84) = 1.298, *p* = 0.273). No individual covariate reached significance: sex (β = 4.26, 95% CI −2.83 to 11.34, *p* = 0.236), dialysis vintage (β = 0.229 per month, −0.244 to 0.701, *p* = 0.339), membrane type (β = −6.31, −14.68 to 2.07, *p* = 0.138), age (β = −0.361 per year, −0.860 to 0.138, *p* = 0.154), and BMI (β = 0.032 per kg/m^2^, −0.641 to 0.704, *p* = 0.926). Multicollinearity was acceptable (all VIF ≤ 4.3). Residuals were non-normal by several tests (e.g., D’Agostino–Pearson K^2^ = 26.95, *p* < 0.0001).

Model 2 (laboratory/inflammation covariates): Overall fit remained modest (R^2^ = 0.081; F (5, 84) = 1.472, *p* = 0.208). Serum creatinine was the only independent predictor of AOPP (β = 2.47 per mg/dL, 95% CI 0.30 to 4.63, *p* = 0.026). eGFR showed a borderline positive association (β = 1.71, −0.23 to 3.65, *p* = 0.084). Albumin (β = −3.06, *p* = 0.515), PC-inflammation (β = 0.747, *p* = 0.306), and CRP (β = 0.031, *p* = 0.865) were not significant. Multicollinearity was low (VIFs ≤ 2.87). Residuals were non-normal (K^2^ = 60.38, *p* < 0.0001).

Interpretation: Models across different covariate sets accounted for approximately 7–8% of the variability in AOPP. After adjustment, higher serum creatinine levels were independently linked to increased AOPP. Other clinical and inflammatory markers, such as the composite PC-inflammation score and CRP, showed no association in this cohort. Due to residual non-normality and a small R^2^, effect sizes should be interpreted with caution; the use of robust or transformed models may be advisable in sensitivity analyses.

##### Multiple Linear Regressions of HD Cohort for TBARS

Two prespecified models were compared. Model 1 used clinical/dialysis covariates (sex, dialysis vintage, membrane type, age, BMI), while Model 2 used laboratory/inflammation covariates (creatinine, albumin, PCA-derived inflammation score, CRP, eGFR). Model 1 was preferred by AICc with an 80.9% probability versus 19.1% for Model 2 (probability ratio 4.231; ΔAICc = −2.885), although overall fit was modest (R^2^ = 0.086, F (5, 84) = 1.587, *p* = 0.173). Within Model 1, longer dialysis vintage was independently associated with lower TBARS (β = −0.2305 per month, 95% CI −0.4253 to −0.0357, *p* = 0.0209); sex, membrane type, age, and BMI were not significant. Model 2 performed worse (R^2^ = 0.056, F (5, 84) = 1.007, *p* = 0.419), and none of its covariates were significant (all *p* ≥ 0.137). Residuals in both models failed normality tests, so effect sizes should be interpreted with caution (Table 8 and Table 9).

#### 2.5.2. Multiple Linear Regressions of T2DM-DN Cohort

##### Multiple Linear Regressions of T2DM-DN Cohort for AOPP

Two prespecified models were compared. Model 1 included demographic and disease–history covariates (sex, years since diabetes diagnosis, age, BMI). Model 2 included laboratory and inflammation covariates (albumin, HbA1c, PCA-derived inflammation score, CRP, eGFR). Model 2 was decisively preferred by AICc (probability 99.9% vs. 0.08% for Model 1; probability ratio 1220; ΔAICc = 14.21), and it explained more variance (R^2^ = 0.214) with a significant overall F test (F(5, 84) = 4.584, *p* = 0.0010). Within Model 2, the PC-inflammation score was the only independent predictor of AOPP (β = 1.134 per unit, 95% CI 0.533–1.734, *p* = 0.0003), while albumin, HbA1c, CRP, and eGFR were not significant. Model 1 fit was weak and non-significant (R^2^ = 0.0556; F(4, 85) = 1.250, *p* = 0.296) and none of its covariates reached significance. Residuals were non-normal in both models, so estimates should be interpreted with appropriate caution or confirmed in sensitivity analyses (Table 10 and Table 11).

##### Multiple Linear Regressions of T2DM-DN Cohort for TBARS

We compared two models. Model 1 used sex, years since diabetes diagnosis, age, and BMI; Model 2 used albumin, HbA1c, PCA-derived inflammation score, CRP, and eGFR. By AICc, Model 1 was preferred (probability 68.98% vs. 31.02% for Model 2; probability ratio 2.224; ΔAICc = −1.598), but both models fit poorly (Table 12 and Table 13).

Model 1: R^2^ = 0.021, overall F (4, 85) = 0.4639, *p* = 0.762. None of the covariates was significant (β [95% CI]; *p*): sex −0.231 [−1.627, 1.165]; *p* = 0.743; years since diagnosis 0.026 [−0.059, 0.111]; *p* = 0.544; age 0.013 [−0.067, 0.094]; *p* = 0.743; BMI −0.056 [−0.190, 0.079]; *p* = 0.413. Residuals failed normality (K^2^ = 56.23, A ^2^* = 7.423, W = 0.739, all *p* < 0.0001).

Model 2: R^2^ = 0.0295, overall F (5, 84) = 0.5114, *p* = 0.767. No covariate reached significance: albumin 0.119 (*p* = 0.881), HbA1c −0.102 (*p* = 0.515), PC-inflammation −0.128 (*p* = 0.446), CRP 0.025 (*p* = 0.641), eGFR 0.020 (*p* = 0.239). Residuals were again non-normal (K^2^ = 52.06, A ^2^* = 7.507, W = 0.745, all *p* < 0.0001).

In T2DM-DN, TBARS were not explained by the tested clinical or laboratory covariates; effect sizes are small and imprecise, and the non-normal residuals argue for cautious interpretation or robust/transform checks.

## 3. Discussion

This study revealed clear differences in how oxidative stress markers (AOPP, TBARS) relate to systemic inflammation in patients with DN versus those on HD. After controlling for age, sex, and serum albumin, the DN group displayed more significant and stable partial correlations, many of which stayed significant after FDR correction. Conversely, the HD group showed fewer and weaker associations after adjustments. These results provide valuable insight into how oxidative stress and inflammation interact across different stages of renal disease.

The stronger oxidative and inflammatory coupling in the DN group suggests that in early or moderate renal impairment, protein oxidation (reflected by AOPP) may act as a key mediator connecting metabolic dysfunction and immune activation. This aligns with mechanistic models of diabetic kidney disease, where hyperglycemia increases ROS formation, activates NADPH oxidases, and upregulates inflammatory cascades (e.g., NF-κB) that mutually amplify ROS production and renal damage [2,3,15]. The continued presence of associations after adjusting for albumin and demographic factors supports the idea that these links are biologically significant rather than influenced by nutritional or demographic differences.

In HD patients, the attenuation of correlations may reflect a more complex milieu. Patients on dialysis are subject to frequent fluctuations in biochemical parameters, interventions such as dialysis vintage, vascular access inflammation, and exposure to bioincompatible membranes. These external perturbations can introduce noise and weaken the detectability of underlying biological relationships. Indeed, oxidative stress in end-stage renal disease is multifactorial, influenced by uremic toxins, the dialysis procedure itself, comorbidities, and antioxidant deficits [1,7,16]. In such settings, a pure oxidative and inflammatory relationship may be overshadowed by episodic events or external stressors, which could explain why fewer associations survived FDR correction in HD.

Moreover, the relative absence of significant TBARS correlations in both groups suggests that lipid peroxidation might play a less consistent role in systemic inflammation, compared to protein oxidation. This differential pattern underscores the possibility that not all oxidative pathways contribute equally to immune activation in renal disease. Markers such as AOPP may better reflect the pathophysiologically relevant oxidative stress in renal patients, especially in DN, where oxidative protein damage has been implicated in glomerular and tubular injury [2,17,18].

In diabetic kidney disease, hyperglycemia induces excessive ROS through activation of NADPH oxidases, mitochondrial dysfunction, and the advanced glycation end products/receptor for AGEs (AGE–RAGE) pathway. This oxidative stress activates NF-κB signaling and cytokine release, including interleukin-6 (IL-6) and tumor necrosis factor-α (TNF-α), promoting chronic inflammation and renal injury. The strong partial correlations between AOPP and inflammation-related indices in our DN group support this mechanistic link, consistent with evidence that oxidative stress and inflammation form a self-amplifying cycle in diabetic nephropathy [2,19].

AOPP are considered more stable indicators of oxidative damage than lipid peroxidation markers like TBARS. AOPP reflect cumulative oxidative modification of plasma proteins and correlate closely with renal function decline and inflammatory cytokine activity [18,20]. The limited associations observed for TBARS in our study are consistent with evidence that the thiobarbituric acid assay lacks specificity and is influenced by multiple reactive carbonyl compounds, making it a less reliable indicator of lipid peroxidation compared to more stable markers such as F2-isoprostanes. According to Ito et al., TBARS can overestimate malondialdehyde levels due to interference from amino acids, bilirubin, and albumin, which may explain the variability and weaker correlations observed in clinical populations [21].

Sources of oxidative stress during HD include exposure to bioincompatible membranes, dialysate contamination, and intermittent ischemia–reperfusion phenomena. Furthermore, accumulation of uremic toxins and repetitive inflammatory stimuli contribute to a persistently activated yet dysregulated immune state [6]. These processes may weaken specific oxidative and inflammatory correlations and produce greater data variability.

In hemodialysis patients, elevated AOPP levels have been linked to chronic microinflammation and endothelial dysfunction, reflecting continuous oxidative stress induced by the dialysis procedure itself rather than metabolic imbalance [7,22,23,24].

Across both cohorts (patients on HD and DN), principal component analysis (PCA) revealed that ten hemogram-derived inflammatory indices grouped into a single latent axis of systemic inflammation (PC-inflammation) with a greater proportion of variance in DN than in HD, reflecting the central role of inflammation and oxidative stress in chronic kidney disease [25]. This pattern aligns with evidence that complete blood count (CBC)-based indices such as the NLR, PLR, SII, and SIRI effectively capture clinically relevant inflammatory burden across CKD severities. SII, in particular, has emerged as a promising biomarker of immune–inflammatory status in end-stage renal disease and hemodialysis populations [26,27,28,29,30].

In HD, AOPP (advanced oxidation protein products) showed an independent association with serum creatinine, a finding biologically plausible given uremic oxidative stress that both produces AOPP and may be further amplified by AOPP in a positive feedback loop. In DN, AOPP were independently linked to the composite PC-inflammation score, strengthening the connection between systemic inflammatory activation and protein oxidation in diabetic kidney disease. Beyond association, recent work suggests AOPP measurement has diagnostic utility for CKD among individuals with T2DM, supporting its translational relevance [9,31,32].

TBARS are widely used as a readout of lipid peroxidation but are method-sensitive and context-dependent, which can dilute associations in multivariable settings [33].

Methodologically, summarizing correlated biomarkers with PCA reduces multicollinearity and distills shared inflammatory variance, a strategy increasingly used in renal omics and cytokine studies, including HD cohorts [34,35].

Clinically, the replication of a one-factor inflammatory structure across HD and DN indicates that a single inflammatory pathway underpins various renal disease contexts; linking this pathway to AOPP in DN and to creatinine in HD suggests shared but cohort-specific oxidative mechanisms that could be targeted with anti-inflammatory and antioxidant therapies [25,32]. Meanwhile, the weaker and context-dependent signal for TBARS demonstrates that lipid peroxidation markers may require prolonged sampling and consideration of dialysis modality, membrane type, and adjunct therapies to establish consistent associations [36,37].

Our pattern shows that AOPP are linked to creatinine in HD and to the PC-inflammation score in DN, with weak or context-sensitive TBARS. This aligns with the review of AN69 membranes, which indicates that more biocompatible, adsorptive dialyzers reduce complement and cytokine activation, thereby decreasing carbonyl and oxidative stress in HD. This diminishes the inflammation’s additional effect on protein oxidation. In contrast, DN without extracorporeal modulation maintains a direct inflammation–protein oxidation pathway [38].

### 3.1. Strengths and Limitations

This study possesses several significant strengths that improve both the reliability and clarity of its results.

First, it offers a comparative analysis between two distinct clinical groups: patients with DN and those undergoing chronic HD. This dual-cohort approach encompasses various stages of renal dysfunction, enabling a direct study of the progression of oxidative and inflammatory interactions from metabolic injury to end-stage renal failure.

Second, the use of partial correlations adjusted for age, sex, and serum ALB reduces bias from key demographic and nutritional confounders known to influence both oxidative and inflammatory biomarkers. Including ALB as a covariate is especially important in nephrology, as hypoalbuminemia indicates both malnutrition and systemic inflammation.

Third, the application of FDR correction strengthens the statistical robustness of the reported associations, ensuring that the observed relationships are less likely to represent chance findings due to multiple testing.

Fourth, evaluating a broad panel of inflammatory indices (AISI, IIC, SII, SIRI, MCVL, NLR, MLR, PLR, dNLR, NPR) together with oxidative stress markers (AOPP, TBARS) provides a multidimensional insight into the redox–immune axis. This comprehensive approach, seldom used in renal populations, offers a wider perspective than studies focusing on a single marker and emphasizes inflammatory pathways linked with oxidative stress, even after adjustments.

Finally, by controlling for major clinical covariates and using quantitative visualization, the study offers a clear, reproducible framework for exploring redox–inflammation relationships in clinical nephrology.

Despite these strengths, some limitations are important to recognize. The cross-sectional design restricts the ability to determine causality; additional longitudinal research is needed to understand whether oxidative stress causes inflammation or is a result of it. While major confounders were accounted for, potential residual confounding from unmeasured factors—such as dialysis membrane composition, dialysate quality, medication use (including antioxidants and erythropoietin), or intercurrent infections—cannot be ruled out.

The sample size, especially in the HD cohort, may limit the power to detect smaller but biologically significant associations and restricts subgroup analyses by sex, dialysis duration, or comorbidity profile. Regarding biomarkers, AOPP and TBARS offer valuable but incomplete insights into oxidative stress pathways, whereas hematologic indices of inflammation are indirect and might not fully capture cytokine-mediated activity.

Finally, the study was conducted in a single-center population, which may limit its applicability to other clinical settings with different patient management strategies or dialysis technologies.

Overall, these strengths and limitations suggest that although the current findings offer a strong basis for understanding the stage-specific oxidative–inflammatory dynamics in kidney disease, they need to be validated with multicenter, long-term studies that include biochemical, cytokine, and metabolomic profiling.

### 3.2. Future Directions

Future research should focus on clarifying the sequence of events and mechanisms connecting oxidative stress and inflammation in the progression of chronic kidney disease. Long-term cohort studies tracking patients from early diabetic nephropathy to dialysis initiation can help determine if changes in oxidative protein damage lead to inflammatory activation or are simply downstream effects of kidney injury. Incorporating multi-omics strategies, such as redox proteomics, lipidomics, and targeted cytokine analysis, can help specify the biochemical nature of these links and uncover causal molecular pathways.

Since oxidative and inflammatory coupling seems strongest in diabetic nephropathy, interventional studies should investigate whether targeted antioxidant or anti-inflammatory treatments (such as NADPH oxidase inhibitors, Nrf2 activators, or advanced glycation end-product (AGE) pathway modulators) can break this feedback loop before permanent renal fibrosis occurs. In hemodialysis, where procedural and metabolic stressors are prevalent, research should focus on dialysis biocompatibility and oxidative load, testing strategies like ultrapure dialysate, vitamin E-coated membranes, and antioxidant supplements to lessen external oxidative stress.

Finally, future clinical translation will rely on developing standardized, reproducible biomarker panels that combine oxidative and inflammatory markers for disease monitoring. Such tools could assist in risk stratification, guiding therapy, and early detection of patients most likely to benefit from targeted redox treatments. Through integrated mechanistic, clinical, and interventional research, the oxidative and inflammatory axis may be transformed from a passive injury marker into an active therapeutic target in kidney disease.

## 4. Materials and Methods

### 4.1. Study Design and Setting

**Figure 3:**
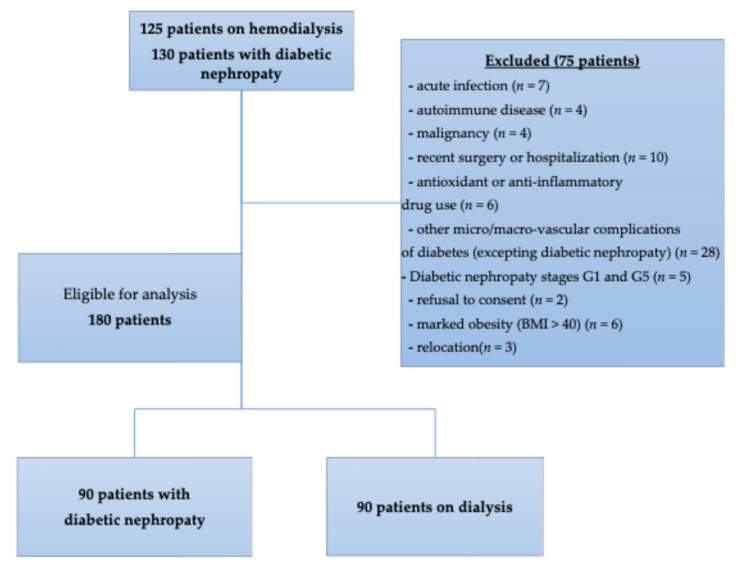
Flowchart of patient inclusion.

This cross-sectional, non-interventional, observational study was conducted over 6 months in the Nephrology Department of the University Hospital of Craiova, Romania, and the Outpatient Diabetes, Nutrition, and Metabolic Diseases Departments of the Filantropia Municipal Clinical Hospital, Craiova, Romania. The research included two patient cohorts: individuals with T2DM-DN and patients undergoing chronic HD. The study aimed to assess the relationship between oxidative stress and systemic inflammation in different stages of renal impairment (Figure 3).

### 4.2. Ethical Considerations

The protocol was approved by the Ethics Committee of the University Hospital of Craiova, Dolj, Romania (approval no. 6/10 January 2025) and by the Ethics Committee of the Filantropia Municipal Clinical Hospital Craiova, Dolj, Romania (approval no. 5024/4 March 2025). All participants provided written informed consent prior to enrollment, in accordance with the ethical principles outlined in the Declaration of Helsinki.

### 4.3. Participants

Eligible participants were adults with confirmed diagnoses of T2DM-DN or end-stage renal disease requiring maintenance hemodialysis for at least 6 months. We enrolled 130 patients with T2DM-DN and 125 patients on HD. Exclusion criteria included acute infection, autoimmune disease, malignancy, recent surgery or hospitalization (<3 months), antioxidant or anti-inflammatory drug use, other micro-/macrovascular complications of diabetes, marked obesity (BMI > 40), relocation, and refusal to consent. After applying the exclusion criteria, both cohorts had a final count of 90 patients. Demographic and clinical data, including age, sex, and BMI were recorded from medical files. The diagnosis of T2DM-DN or end-stage renal disease requiring maintenance hemodialysis were in accordance with American Diabetes Association (ADA), Kidney Disease: Improving Global Outcomes (KDIGO) [39,40]. Patients with T2DM-DN that were enrolled and classified into stages between G2 and G4 conform to KDIGO staging.

### 4.4. Evaluation of Inflammatory Status

In order to evaluate the inflammatory status, we utilized the formulas from Table 14.

Furthermore, eGFR was determined utilizing the serum creatinine-based CKD-EPI equation, as operationalized within the validated MDCalc calculator [45].

### 4.5. Sampling Procedure

During biological sampling, venous blood was drawn from each participant using two types of vacutainer tubes. For patients on ongoing HD, samples were collected before the procedure. Two additive-free tubes (Becton Dickinson Vacutainer, Franklin Lakes, NJ, USA), each holding roughly 5 mL of blood, were used for serum analyses. The samples were allowed to clot at room temperature and processed within four hours of collection. Centrifugation was carried out at 3000× *g* for 10 min using a Hermle centrifuge (Hermle AG, Gosheim, Germany).

The serum was meticulously separated to reduce hemolysis. A portion was divided into pre-labeled cryovials, hermetically sealed, and stored at temperatures from −20 °C to −80 °C to preserve protein integrity. Freeze–thaw cycles were strictly avoided. Before analysis, aliquots were brought to room temperature under controlled conditions. One aliquot was set aside for immunological assays, and the serum from the second vial was reserved for biochemical analyses.

For hematological analysis, a third blood sample was collected in an EDTA vacutainer tube (Becton Dickinson, Franklin Lakes, NJ, USA).

### 4.6. Laboratory Assays

Serum biochemical parameters were measured using the ARCHITECT C4000 automated chemistry analyzer (Abbott Diagnostics, Abbott Park, IL, USA), following standardized clinical laboratory protocols to ensure accuracy and reproducibility.

A comprehensive leukocyte differential count was performed using the MINDRAY BC-6800 hematology analyzer (Mindray, Shenzhen, China). This system combines flow cytometry and impedance technology (Coulter principle) to accurately identify and count leukocyte subsets. The method provides precise measurement of neutrophils, lymphocytes, monocytes, eosinophils, and basophils, offering a detailed leukocyte profile and supporting the calculation of inflammatory indices for further analysis.

### 4.7. Enzyme-Linked Immunosorbent Assay of AOPP and TBARS

At the Immunology Laboratory of the University of Medicine and Pharmacy of Craiova, serum concentrations of AOPP and TBARS were measured using the enzyme-linked immunosorbent assay (ELISA) method. We followed the protocol instructions to determine serum levels of AOPP and TBARS, employing ELISA kits from MyBioSource (San Diego, CA, USA) and using a standard optical analyzer set at a wavelength of 450 nm for the process (Table 15).

### 4.8. Statistical Assessment

The patient data extracted from medical records were systematically organized and curated using Microsoft Excel. Statistical analyses were conducted with GraphPad Prism 10.6.1 (GraphPad Software, San Diego, CA, USA) and R v4.3.2. The data distribution’s normality was assessed using the Shapiro–Wilk and D’Agostino’s tests. Parametric data were analyzed using Welch’s *t*-test, whereas non-parametric data were evaluated with the Mann–Whitney U test. Categorical variables are presented as percentages to depict frequency distributions. Partial correlation analyses were conducted to explore the relationships between oxidative stress markers (AOPP and TBARS) and inflammatory indices (AISI, IIC, SII, SIRI, MCVL, NLR, MLR, PLR, dNLR, NPR) while adjusting for age, sex, and serum ALB. The Benjamini–Hochberg FDR method was used to control for multiple testing. A two-tailed *p*-value < 0.05 was considered statistically significant before correction, and a q-value < 0.05 was considered significant after FDR adjustment. Values with 0.05 ≤ q < 0.10 were interpreted as trends toward significance. For MLR, normality of Residuals was assessed with: D’Agostino-Pearson omnibus (K^2^), Anderson-Darling (A ^2^*), Shapiro-Wilk (W).

## 5. Conclusions

AOPP and TBARS were markedly higher in hemodialysis than in diabetic nephropathy, supporting a stronger oxidative burden in end-stage disease. Both differences were large and statistically significant.

In diabetic nephropathy, AOPP showed consistent positive correlations with multiple inflammatory indices after adjustment and false discovery rate correction. This pattern indicates tight coupling between protein oxidation and systemic inflammation in this group. TBARS showed no adjusted associations. In hemodialysis, correlations between oxidative markers and inflammatory indices did not remain significant after correction. This suggests that dialysis-related factors and uremic toxicity weaken the oxidative inflammation link.

Principal-component analysis supported a single latent inflammatory axis in both cohorts, stronger in diabetic nephropathy. In regression models, AOPP associated independently with creatinine in hemodialysis and with the composite inflammation score in diabetic nephropathy. AOPP are a practical marker to track the inflammation related oxidative signal in diabetic nephropathy. Their association with creatinine in hemodialysis points to uremic load and dialysis milieu rather than inflammatory activation. These insights can guide monitoring, for example, pairing AOPP with CBC-derived indices in diabetic nephropathy and targeting dialysis biocompatibility and uremic control in hemodialysis.

## Figures and Tables

**Table 1:** Demographic, clinical, and paraclinical characteristics.

Parameter	HD 90 Patients	T2DM-DN 90 Patients	*p*-Value
**Age (years)** **[median (range)]**	61 (23–89)	67 (40–83)	0.03 *
**Sex (n)** **Male/Female**	43/47	48/42	0.454
**Residence (n)** **Urban/Rural**	24/66	60/30	<0.0001 *
**Weight (kg)** **[median (range)]**	74.25 (39–103)	84.50 (45–137)	<0.0001 *
**Height (m)** **[median (range)]**	1.66 (1.19–1.98)	1.68 (1.47–1.89)	0.281
**BMI (kg/m^2^)** **[median (range)]**	26.40 (17.33–35.43)	31.11 (20.82–45.55)	<0.0001 *
**TG (mg/dL)** **[median (range)]**	133 (49–401)	145 (57–397)	0.668
**TC (mg/dL)** **[median (range)]**	171.50 (92–291)	162 (84.00–364)	0.777
**WBC (×10^3^/μL)** **[median (range)]**	6.77 (2.72–10.30)	8.42 (4.90–25.36)	<0.0001 *
**NEU (×10^3^/μL)** **[median (range)]**	4.20 (1.12–7.20)	5.23 (2.68–23.67)	<0.0001 *
**LYM (×10^3^/μL)** **[median (range)]**	1.59 (0.20–3.22)	1.92 (0.28–6.05)	0.0005 *
**BAS (×10^3^/μL)** **[median (range)]**	0.05 (0.00–0.17)	0.04 (0.01–0.40)	0.0006 *
**EOS (×10^3^/μL)** **[median (range)]**	0.20 (0.00–0.82)	0.16 (0.01–0.76)	0.012 *
**MON (×10^3^/μL)** **[median (range)]**	0.60 (0.31–1.77)	0.58 (0.20–1.19)	0.211
**RBC (×10^3^/μL)** **(Mean ± SD)**	3.60 ± 0.54	4.21 ± 0.67	<0.0001 *
**HCT (%)** **(Mean ± SD)**	34.21 ± 4.57	37.42 ± 6.71	0.0003 *
**HGB (g/dL)** **[median (range)]**	10.90 (7.90–14.20)	12.65 (8.10–17.30)	<0.0001 *
**MCV (fL)** **[median (range)]**	96.60 (64.60–125.00)	89.35 (69.00–106.30)	<0.0001 *
**PLT (×10^3^/μL)** **[median (range)]**	192.00 (57.20–438.00)	240.50 (116.00–573.00)	<0.0001 *
**Creatinine (mg/dL)** **[median (range)]**	8.81 (3.25–16.00)	1.09 (0.59–2.83)	<0.0001 *
**Urea (mg/dL)** **[median (range)]**	135.90 (36.38–387.30)	52.00 (24.00–159.00)	<0.0001 *
**eGFR (mL/min/1.73 m^2^)** **CKD-EPI** **[median (range)]**	5.51 (2.14–20.81)	57.39 (16.55–89.96)	<0.0001 *
**Na** **(Mean ± SD)**	138.6 ± 2.90	137.90 ± 4.04	0.198
**K** **[median (range)]**	4.80 (2.50–7.80)	4.55 (3.10–7)	0.006 *
**Cl** **[median (range)]**	105.50 (99.00–122.00)	102 (90.00–110)	<0.0001 *
**ALT (mg/dL)** **[median (range)]**	16 (6.00–150)	22 (7.00–136)	0.001 *
**AST (mg/dL)** **[median (range)]**	16 (4.00–186)	21.39 (9.85–65.43)	<0.0001 *
**Hemodialysis Vintage** **(months)**	48 ± 22	-	-
**Membrane type** **(Synthetic high-flux/Synthetic low-flux) (n/%)**	-	77/13 85.56%/14.44%	-
**Years of diagnosis** **(years)**	-	12.06 ± 8.23	-
**HbA1c (%)**	-	9.57 ± 2.19	-

Demographic, anthropometric, hematological, renal, and biochemical characteristics of patients with hemodialysis (HD) and type 2 diabetes mellitus with diabetic nephropathy (T2DM-DN). Data are presented as median (range) or mean ± standard deviation, as appropriate. Significant differences (*p* < 0.05) indicate distinct metabolic and renal profiles between groups, reflecting advanced renal dysfunction in HD and higher metabolic burden in T2DM-DN. BMI: body mass index; TG: triglycerides; TC: total cholesterol; WBC: white blood cells; NEU: neutrophils; MON: monocytes; LYM: lymphocytes; EOS: eosinophils; BAS: basophils; RBC: red blood cells; HCT: hematocrit; HGB: hemoglobin; MCV: mean corpuscular volume; PLT: plaquettes; eGFR: estimated glomerular filtration rate; CKD-EPI: Chronic Kidney Disease Epidemiology Collaboration; ALT: alanine transaminase; AST: aspartate aminotransferase; Na: Sodium; K: Potassium; Cl: Chloride; *: reached the significant differences (*p* < 0.05).

**Table 2:** Levels of AOPP, TBARS, and inflammatory status.

Parameter	HD	T2DM-DN	*p*-Value	Parameter	HD	T2DM-DN	*p*-Value
**CRP (mg/dL)** **[median (range)]**	4.50 (0.30–49.40)	1.08 (0.02–30.42)	<0.0001 *	**AISI** **[median (range)]**	317.30 (24.98–6087)	355.80 (69.36–4707)	0.09
**ALB (g/dL)** **[median (range)]**	4.10 (3.20–4.80)	4.40 (3.50–5.30)	<0.0001 *	**SIRI** **[median (range)]**	1.67 (0.30–21.97)	1.43 (0.32–39.47)	0.339
**IIC** **[median (range)]**	3.72 (1.44–34.66)	3.00 (1.19–59.68)	0.02 *	**SII** **[median (range)]**	505.20 (77.83–6745)	603.30 (192.70–6272)	0.002 *
**NPR** **[median (range)]**	22.25 (9.59–72.99)	21.75 (8.67–130.50)	0.775	**NLR** **[median (range)]**	2.57 (0.95–24.35)	2.56 (1.00–54.07)	0.06
**MCVL** **[median (range)]**	61.42 (23.99–434)	46.28 (13.60–315.40)	<0.0001 *	**MLR** **[median (range)]**	0.39 (0.11–4.51)	0.28 (0.10–2.61)	<0.0001 *
**AOPP** **[median (range)]**	25.80 (2.48–50)	5.06 (2.29–25.79)	<0.0001 *	**PLR** **[median (range)]**	128.40 (45.32–1385)	124.70 (44.96–521.90)	0.485
**TBARS** **[median (range)]**	8.49 (0.53–29.94)	1.89 (0.31–15.78)	<0.0001 *	**dNLR** **[median (range)]**	1.66 (0.70–4.25)	1.81 (0.86–14.56)	0.03 *

Inflammatory and oxidative stress parameters in HD and T2DM-DN patients. Variables include systemic inflammation indices (AISI, IIC, SII, SIRI, NLR, MLR, PLR, dNLR, NPR) and oxidative stress markers (AOPP, TBARS). Data are expressed as median (range). HD patients exhibited higher CRP, AOPP, TBARS, and MCVL levels, indicating a more pronounced oxidative and inflammatory state, while T2DM-DN patients showed higher SII values, reflecting metabolic inflammation. *: reached the significant differences (*p* < 0.05); AOPP: advanced oxidation protein products; TBARS: thiobarbituric acid reactive substances; ALB: albumin; CRP: C-reactive protein; AISI: aggregate index of systemic inflammation; IIC: immune inflammation coefficient; SII: systemic immune–inflammation index; SIRI: systemic inflammation response index; MCVL: ratio between mean corpuscular volume and lymphocytes; NLR: neutrophil-to-lymphocyte ratio; MLR: monocyte-to-lymphocyte ratio; PLR: platelet-to-lymphocyte ratio; dNLR: derived neutrophil-to-lymphocyte ratio; NPR: neutrophil-to-platelet ratio.

**Table 3:** Partial correlations of AOPP and TBARS and inflammatory status.

Parameter		Number	r-Value	*p*-Value	q-Value	Parameter		Number	r-Value	*p*-Value	q-Value
*HD*											
AOPP	AISI	90	−0.018	0.864	0.911	TBARS	AISI	90	0.083	0.439	0.798
	IIC	90	0.148	0.167	0.798		IIC	90	0.041	0.704	0.911
	SII	90	0.036	0.741	0.911		SII	90	0.085	0.431	0.798
	SIRI	90	0.043	0.691	0.911		SIRI	90	0.019	0.863	0.911
	MCVL	90	0.238	0.024 *	0.489		MCVL	90	−0.015	0.886	0.911
	NLR	90	0.147	0.170	0.798		NLR	90	0.019	0.862	0.911
	MLR	90	0.085	0.428	0.798		MLR	90	−0.012	0.912	0.911
	PLR	90	0.089	0.407	0.798		PLR	90	0.067	0.530	0.882
	dNLR	90	0.131	0.219	0.798		dNLR	90	0.092	0.389	0.798
	NPR	90	0.086	0.423	0.798		NPR	90	−0.141	0.187	0.798
*T2DM-DN*											
AOPP	AISI	90	0.325	0.001 *	0.004	TBARS	AISI	90	0.018	0.864	0.882
	IIC	90	0.428	<0.0001 *	0.0002		IIC	90	−0.060	0.574	0.882
	SII	90	0.384	0.0002 *	0.0006		SII	90	−0.021	0.847	0.882
	SIRI	90	0.391	0.0001 *	0.0005		SIRI	90	−0.028	0.797	0.882
	MCVL	90	0.401	0.0001 *	0.0004		MCVL	90	−0.033	0.760	0.882
	NLR	90	0.429	<0.0001 *	0.0002		NLR	90	−0.055	0.607	0.882
	MLR	90	0.342	0.001 *	0.002		MLR	90	0.016	0.882	0.882
	PLR	90	0.248	0.018 *	0.03		PLR	90	0.018	0.868	0.882
	dNLR	90	0.404	0.0001 *	0.0004		dNLR	90	−0.039	0.719	0.882
	NPR	90	0.353	0.0007 *	0.001		NPR	90	−0.075	0.482	0.876

Partial correlations between oxidative stress markers (AOPP and TBARS) and inflammatory indices in HD and T2DM-DN patients after adjustment for age, sex, and serum ALB. Correlation coefficients (r), unadjusted *p*-values, and false discovery rate-adjusted q-values (Benjamini–Hochberg correction) are presented. In the T2DM-DN group, AOPP shows moderate positive correlations with multiple inflammatory markers (IIC, SII, SIRI, MCVL, NLR, MLR, PLR, dNLR, and NPR), indicating a robust link between protein oxidation and systemic inflammation. No correlations remained significant after adjustment in the HD group, suggesting that oxidative and inflammatory coupling weakens in advanced renal failure. *: reached the significant differences (*p* < 0.05)

**Table 4:** Eigenvalues and variance explained for each component, PC1 to PC10, with cumulative variance.

Component	Eigenvalue	Variance Explained (%)	Cumulative Proportion of Variance (%)	Component	Eigenvalue	Variance Explained (%)	Cumulative Proportion of Variance (%)
*HD*				*T2DM-DN*			
**PC1**	7.473	74.73%	74.73%	**PC1**	8.52	85.20%	85.20%
**PC2**	1.386	13.86%	88.60%	**PC2**	0.7327	7.33%	92.52%
**PC3**	0.6726	6.73%	95.32%	**PC3**	0.3636	3.64%	96.16%
**PC4**	0.3574	3.57%	98.90%	**PC4**	0.3053	3.05%	99.21%
**PC5**	0.053	0.53%	99.43%	**PC5**	0.0428	0.43%	99.64%
**PC6**	0.0297	0.30%	99.72%	**PC6**	0.0161	0.16%	99.80%
**PC7**	0.0169	0.17%	99.89%	**PC7**	0.0126	0.13%	99.93%
**PC8**	0.0076	0.08%	99.97%	**PC8**	0.0056	0.06%	99.98%
**PC9**	0.0019	0.02%	99.99%	**PC9**	0.0011	0.01%	99.99%
**PC10**	0.0012	0.01%	100.00%	**PC10**	0.0007	0.01%	100.00%

Eigenvalues and variance explained by principal components in the HD and DN cohorts. The table lists eigenvalues for PC1 to PC10, percent variance, and cumulative percent variance, separately for each cohort. Variables were z standardized, and PCA used the correlation matrix. Parallel analysis with 1000 simulations guided component retention. Each cohort contributed 90 complete cases.

**Table 5:** Loadings of the ten indices on PC1.

Variable	AISI	IIC	SII	SIRI	MCVL	NLR	MLR	PLR	dNLR	NPR
**PC1 loading-HD**	−0.9415	−0.9728	−0.9769	−0.9674	−0.8821	−0.9819	−0.9558	−0.9731	−0.3762	0.0798
**PC1 loading-T2DM-DN**	−0.9149	−0.9791	−0.9647	−0.9653	−0.9095	−0.9783	−0.924	−0.7565	−0.9409	−0.8751

PC1 loadings of the ten inflammatory indices in the HD and DN cohorts. The table reports loadings for AISI, IIC, SII, SIRI, MCVL, NLR, MLR, PLR, dNLR, and NPR, estimated from PCA of the correlation matrix with z standardized inputs. Loadings quantify the strength and direction of association with PC1, higher absolute values indicate stronger contributions. The sign of loadings is arbitrary and does not affect interpretation. Each cohort included 90 complete cases.

**Table 6:** Model comparison and analysis of variance for multiple linear regression of AOPP in hemodialysis patients.

	Analysis of Variance	SS	DF	MS	F (DFn, DFd)	*p* Value
**Model 1**	Regression	1743	5	348.7	F (5, 84) = 1.298	0.2728
	Sex-HD	383.4	1	383.4	F (1, 84) = 1.426	0.2357
	HD vintage	248.3	1	248.3	F (1, 84) = 0.9240	0.3392
	Membrane-HD	602.8	1	602.8	F (1, 84) = 2.243	0.1380
	Age-HD	555.3	1	555.3	F (1, 84) = 2.066	0.1543
	BMI-HD	2.34	1	2.34	F (1, 84) = 0.008706	0.9259
**Model 2**	Regression	1958	5	391.7	F (5, 84) = 1.472	0.2078
	Creatinine-HD	1366	1	1366	F (1, 84) = 5.131	0.0261
	ALB-HD	114.1	1	114.1	F (1, 84) = 0.4286	0.5145
	PC-inflammation	282.3	1	282.3	F (1, 84) = 1.061	0.3060
	CRP-HD	7.803	1	7.803	F (1, 84) = 0.02932	0.8645
	eGFR-HD	816.1	1	816.1	F (1, 84) = 3.066	0.0836

Two prespecified models were evaluated. Model 1 covariates: sex, dialysis vintage, membrane type, age, BMI. Model 2 covariates: creatinine, albumin, PCA-derived inflammation score (PC-inflammation), CRP, eGFR. The table reports SS, DF, MS, F, and two-sided *p* for the overall model and for each term; AICc model comparison favored Model 2 (probability 0.606 vs. 0.394; ΔAICc = 0.861). Residual df = 84. Abbreviations: AOPP, advanced oxidation protein products; HD, hemodialysis; BMI, body mass index; CRP, C-reactive protein; eGFR, estimated glomerular filtration rate. Overall fits were modest and not significant (Model 1 F (5, 84) = 1.298, *p* = 0.2728; Model 2 F (5, 84) = 1.472, *p* = 0.2078); AICc provided only weak preference for Model 2 over Model 1. SS: Sum of Squares; DF: Degrees of Freedom; MS: Mean Square; DFn: numerator DF; DFd: denominator DF.

**Table 7:** Parameter estimates for multiple linear regression of AOPP in hemodialysis patients.

		Variable	Estimate	95% CI (Profile Likelihood)	|t|	*p* Value
**Model 1**	β0	Intercept	40.55	18.11 to 62.98	3.594	0.0005
	β1	Sex-HD	4.256	−2.830 to 11.34	1.194	0.2357
	β2	HD vintage	0.2286	−0.2443 to 0.7014	0.9613	0.3392
	β3	Membrane-HD	−6.305	−14.68 to 2.066	1.498	0.138
	β4	Age-HD	−0.3607	−0.8598 to 0.1383	1.438	0.1543
	β5	BMI-HD	0.03156	−0.6411 to 0.7042	0.09331	0.9259
**Model 2**	β0	Intercept	10.15	−40.08 to 60.38	0.4017	0.6889
	β1	Creatinine-HD	2.467	0.3011 to 4.632	2.265	0.0261
	β2	ALB-HD	−3.055	−12.34 to 6.226	0.6547	0.5145
	β3	PC-inflammation	0.7471	−0.6956 to 2.190	1.03	0.306
	β4	CRP-HD	0.03098	−0.3288 to 0.3908	0.1712	0.8645
	β5	eGFR-HD	1.708	−0.2318 to 3.648	1.751	0.0836

Unstandardized coefficients (β), profile-likelihood 95% CIs, |t|, and two-sided *p* values are shown for both models. In Model 1, none of the clinical/dialysis covariates was significant. In Model 2, creatinine was the only independent predictor of AOPP (β = 2.467 per mg/dL, 95% CI 0.301–4.632, *p* = 0.026), while albumin, PC-inflammation, CRP, and eGFR were not significant (eGFR borderline, *p* = 0.084). |t|, absolute Student’s t statistic (|β/SE|; degrees of freedom = residual df); PC-inflammation is the first principal-component score summarizing the 10 inflammatory indices.

**Table 8:** Model comparison and analysis of variance for multiple linear regression of TBARS in hemodialysis patients.

	Analysis of Variance	SS	DF	MS	F (DFn, DFd)	*p* Value
**Model 1**	Regression	361.8	5	72.37	F (5, 84) = 1.587	0.1727
	Sex-HD	25.99	1	25.99	F (1, 84) = 0.5699	0.4524
	HD vintage	252.6	1	252.6	F (1, 84) = 5.538	0.0209
	Membrane-HD	0.09155	1	0.09155	F (1, 84) = 0.002007	0.9644
	Age-HD	118.8	1	118.8	F (1, 84) = 2.604	0.1103
	BMI-HD	1.297	1	1.297	F (1, 84) = 0.02844	0.8665
**Model 2**	Regression	237	5	47.41	F (5, 84) = 1.007	0.4190
	Creatinine-HD	0.06341	1	0.06341	F (1, 84) = 0.001346	0.9708
	ALB-HD	106.2	1	106.2	F (1, 84) = 2.256	0.1369
	PC-inflammation	5.306	1	5.306	F (1, 84) = 0.1127	0.7380
	CRP-HD	75.69	1	75.69	F (1, 84) = 1.607	0.2084
	eGFR-HD	21.49	1	21.49	F (1, 84) = 0.4564	0.5012

Two models were evaluated. Model 1 covariates: sex, dialysis vintage, membrane type, age, BMI. Model 2 covariates: creatinine, albumin, PCA-derived inflammation score (PC-inflammation), CRP, eGFR. The table reports SS, DF, MS, F, and two-sided *p* for the overall model and each term, plus AICc model comparison statistics (Model 1 probability 80.88%, Model 2 probability 19.12%, probability ratio 4.231, ΔAICc −2.885; residual df 84). TBARS, thiobarbituric acid reactive substances; HD, hemodialysis; BMI, body mass index; CRP, C-reactive protein; eGFR, estimated glomerular filtration rate.

**Table 9:** Parameter estimates for multiple linear regression of TBARS in hemodialysis patients.

		Variable	Estimate	95% CI (Profile Likelihood)	|t|	*p* Value
**Model 1**	β0	Intercept	10.87	1.627 to 20.11	2.339	0.0217
	β1	Sex-HD	1.108	−1.811 to 4.028	0.7549	0.4524
	β2	HD vintage	−0.2305	−0.4253 to −0.03572	2.353	0.0209
	β3	Membrane-HD	0.0777	−3.371 to 3.526	0.0448	0.9644
	β4	Age-HD	0.1668	−0.03876 to 0.3724	1.614	0.1103
	β5	BMI-HD	0.0235	−0.2536 to 0.3006	0.1686	0.8665
**Model 2**	β0	Intercept	−2.857	−23.99 to 18.27	0.2689	0.7887
	β1	Creatinine-HD	0.01681	−0.8940 to 0.9276	0.03669	0.9708
	β2	ALB-HD	2.948	−0.9556 to 6.852	1.502	0.1369
	β3	PC-inflammation	0.1024	−0.5044 to 0.7093	0.3357	0.738
	β4	CRP-HD	−0.0965	−0.2478 to 0.05486	1.268	0.2084
	β5	eGFR-HD	0.2772	−0.5388 to 1.093	0.6756	0.5012

Unstandardized coefficients (β), profile-likelihood 95% CIs, |t|, and two-sided *p* values are shown for each model. In Model 1, only dialysis vintage was significant (β = −0.2305 per month, 95% CI −0.4253 to −0.0357, *p* = 0.0209). In Model 2, creatinine, albumin, PC-inflammation, CRP, and eGFR were not significant. PC-inflammation is the first principal-component score summarizing the 10 inflammatory indices.

**Table 10:** Model comparison and analysis of variance for multiple linear regression of AOPP in diabetic nephropathy.

	Analysis of Variance	SS	DF	MS	F (DFn, DFd)	*p* Value
**Model 1**	Regression	198.3	4	49.57	F (4, 85) = 1.250	0.2960
	Sex-DN	2.672	1	2.672	F (1, 85) = 0.06739	0.7958
	Years of diagnosis-DN	20.49	1	20.49	F (1, 85) = 0.5167	0.4742
	Age-DN	87.12	1	87.12	F (1, 85) = 2.197	0.1419
	BMI-DN	92.49	1	92.49	F (1, 85) = 2.333	0.1304
**Model 2**	Regression	764.9	5	153	F (5, 84) = 4.584	0.0010
	ALB-DN	29.13	1	29.13	F (1, 84) = 0.8727	0.3529
	HbA1c-DN	59.15	1	59.15	F (1, 84) = 1.772	0.1867
	PC-inflammation	470.5	1	470.5	F (1, 84) = 14.10	0.0003
	CRP-DN	0.04513	1	0.04513	F (1, 84) = 0.001352	0.9708
	eGFR-DN	0.6729	1	0.6729	F (1, 84) = 0.02016	0.8874

Two models were evaluated. Model 1 covariates: sex, years since diabetes diagnosis, age, BMI. Model 2 covariates: albumin, HbA1c, PCA-derived inflammation score (PC-inflammation), CRP, eGFR. Shown are SS, DF, MS, F, and two-sided *p* for the overall model and each term, plus AICc model comparison (Model 2 probability 99.92%, Model 1 probability 0.0819%, probability ratio 1220, ΔAICc 14.21; residual df 84–85). Abbreviations: AOPP, advanced oxidation protein products; DN, diabetic nephropathy; BMI, body mass index; CRP, C-reactive protein; eGFR, estimated glomerular filtration rate. Model 2 fit the data significantly better; Model 1 showed a poor fit and no significant terms.

**Table 11:** Parameter estimates for multiple linear regression of AOPP in diabetic nephropathy.

		Variable	Estimate	95% CI (Profile Likelihood)	|t|	*p* Value
**Model 1**	β0	Intercept	8.702	−4.840 to 22.24	1.278	0.2049
	β1	Sex-DN	−0.3596	−3.114 to 2.394	0.2596	0.7958
	β2	Years of diagnosis-DN	0.06061	−0.1070 to 0.2282	0.7188	0.4742
	β3	Age-DN	−0.118	−0.2764 to 0.04029	1.482	0.1419
	β4	BMI-DN	0.2035	−0.06140 to 0.4683	1.527	0.1304
**Model 2**	β0	Intercept	3.975	−10.43 to 18.38	0.5487	0.5847
	β1	ALB-DN	1.342	−1.514 to 4.197	0.9342	0.3529
	β2	HbA1c-DN	−0.3768	−0.9396 to 0.1860	1.331	0.1867
	β3	PC-inflammation	1.134	0.5333 to 1.734	3.755	0.0003
	β4	CRP-DN	0.0035	−0.1856 to 0.1926	0.03677	0.9708
	β5	eGFR-DN	−0.0042	−0.06343 to 0.05498	0.142	0.8874

Unstandardized coefficients (β), profile-likelihood 95% CIs, |t|, and two-sided *p* values are reported for both models. In Model 2, PC-inflammation independently predicted higher AOPP (β = 1.134, 95% CI 0.533–1.734, *p* = 0.0003); albumin, HbA1c, CRP, and eGFR were not significant. In Model 1, sex, years since diagnosis, age, and BMI were not significant; PC-inflammation is the first principal-component score summarizing the ten inflammatory indices.

**Table 12:** Model comparison and analysis of variance for multiple linear regression of TBARS in diabetic nephropathy.

	Analysis of Variance	SS	DF	MS	F (DFn, DFd)	*p* Value
**Model 1**	Regression	18.9	4	4.725	F (4, 85) = 0.4639	0.7620
	Sex-DN	1.102	1	1.102	F (1, 85) = 0.1082	0.7431
	Years of diagnosis-DN	3.779	1	3.779	F (1, 85) = 0.3710	0.5441
	Age-DN	1.106	1	1.106	F (1, 85) = 0.1085	0.7426
	BMI-DN	6.9	1	6.9	F (1, 85) = 0.6774	0.4128
**Model 2**	Regression	26.14	5	5.227	F (5, 84) = 0.5114	0.7669
	ALB-DN	0.2306	1	0.2306	F (1, 84) = 0.02256	0.8810
	HbA1c-DN	4.367	1	4.367	F (1, 84) = 0.4273	0.5151
	PC-inflammation	6.003	1	6.003	F (1, 84) = 0.5873	0.4456
	CRP-DN	2.234	1	2.234	F (1, 84) = 0.2186	0.6413
	eGFR-DN	14.37	1	14.37	F (1, 84) = 1.406	0.2391

Two prespecified OLS models were evaluated. Model 1 covariates: sex, years since diabetes diagnosis, age, BMI. Model 2 covariates: albumin, HbA1c, PCA-derived inflammation score (PC-inflammation), CRP, eGFR. The table lists SS, DF, MS, F and two-sided *p* for the overall model and each term, alongside AICc model comparison (Model 1 probability 68.98%, Model 2 probability 31.02%, probability ratio 2.224, ΔAICc −1.598; residual df 84–85). Abbreviations: TBARS, thiobarbituric acid reactive substances; DN, diabetic nephropathy; BMI, body mass index; CRP, C-reactive protein; eGFR, estimated glomerular filtration rate.

**Table 13:** Parameter estimates for multiple linear regression of TBARS in diabetic nephropathy.

		Variable	Estimate	95% CI (Profile Likelihood)	|t|	*p* Value
**Model 1**	β0	Intercept	3.67	−3.193 to 10.53	1.063	0.2907
	β1	Sex-DN	−0.2309	−1.627 to 1.165	0.3289	0.7431
	β2	Years of diagnosis-DN	0.02603	−0.05894 to 0.1110	0.6091	0.5441
	β3	Age-DN	0.0133	−0.06696 to 0.09355	0.3295	0.7426
	β4	BMI-DN	−0.0556	−0.1898 to 0.07867	0.823	0.4128
**Model 2**	β0	Intercept	2.338	−5.635 to 10.31	0.5831	0.5614
	β1	ALB-DN	0.1194	−1.461 to 1.700	0.1502	0.881
	β2	HbA1c-DN	−0.1024	−0.4139 to 0.2091	0.6537	0.5151
	β3	PC-inflammation	−0.1281	−0.4604 to 0.2042	0.7663	0.4456
	β4	CRP-DN	0.0246	−0.08005 to 0.1293	0.4675	0.6413
	β5	eGFR-DN	0.01953	−0.01323 to 0.05230	1.186	0.2391

Unstandardized coefficients (β), profile-likelihood 95% CIs, |t|, and two-sided *p* values are presented for each model. None of the predictors reached statistical significance in either model. PC-inflammation is the first principal-component score summarizing the ten inflammatory indices.

**Table 14:** Formulas utilized in the evaluation of inflammatory status [41,42,43,44].

Index	Formula
**AISI**	((neutrophils × monocytes × platelets)/lymphocytes)
**IIC**	((mean corpuscular volume × width of erythrocyte distribution × neutrophils)/(lymphocytes × 1000))
**SII**	(neutrophils × platelets)/lymphocytes
**SIRI**	((neutrophil × monocytes)/lymphocytes)
**MCVL**	mean corpuscular volume/lymphocytes
**NLR**	neutrophil-to-lymphocyte ratio
**MLR**	monocyte-to-lymphocyte ratio
**PLR**	platelet-to-lymphocyte ratio
**dNLR**	neutrophils/(leucocytes − neutrophils)
**NPR**	(neutrophil counts × 1000)/platelet counts

**Table 15:** Main characteristics of the ELISA kits [46,47].

	AOPP	TBARS
**Catalog Number**	MBS722252	MBS166987
**Sensitivity**	0.1 ng/mL	0.022 nmol/mL
**Detection range**	2.5–50 ng/mL	0.05–30 nmol/mL
**Cross reactivity**	No significant cross-reactivity or interference between AOPP and analogs was observed.	-
**Intra-/Inter-assay CV (%)**	CV < 10%; CV < 12%	CV < 8%; CV < 10%
**ISO Certification**	Manufactured in an ISO 13485:2016 Certified Laboratory.	Manufactured in an ISO 9001:2015 Certified Laboratory.
**Assay Type**	Competitive	Quantitative Sandwich
**Spike Recovery**	92–101%	-
**Sample required/well**	40 microL	40 microL

## Data Availability

The data used to support the findings of this study are available from the corresponding author upon reasonable request. The data are not publicly available due to ethical reasons.
